# Analysis of mating system, fecundity, hatching and survival rates in two *Schistosoma mansoni* intermediate hosts (*Biomphalaria pfeifferi* and *Biomphalaria camerunensis*) in Cameroon

**DOI:** 10.1186/s13071-015-1285-4

**Published:** 2016-01-06

**Authors:** Alvine C. Kengne-Fokam, Hugues C. Nana-Djeunga, Félicité F. Djuikwo-Teukeng, Flobert Njiokou

**Affiliations:** University of Yaoundé 1, Parasitology and Ecology Laboratory, Faculty of Science, PO Box 812, Yaoundé, Cameroon; Centre for Research on Filariasis and other Tropical Diseases (CRFilMT), PO Box 5797, Yaoundé, Cameroon; Université des Montagnes, PO Box 208, Bangangté, Cameroon

**Keywords:** Mating system, *Biomphalaria pfeifferi*, *Biomphalaria camerunensis*, Life history traits, Inbreeding depression, *Schistosoma mansoni*

## Abstract

**Background:**

*Biomphalaria pfeifferi* and *Biomphalaria camerunensis* are intermediate hosts of the trematode *Schistosoma mansoni*. Up till now, very scanty data report the life history traits of these freshwater snails. This study was therefore conducted to provide further knowledge on the mating system of these two *S. mansoni* intermediate hosts in Cameroon. The study was performed following a three-step experimental design as follows: (i) for each species, a sample of young snails (G_1_), virgin and sexually mature was constituted and divided into two groups; (ii) in the first group, individuals were maintained isolated for the evaluation of the impact of self-fertilization on life history traits while in the second group, individuals were paired for few hours for the evaluation of cross-fertilization impact; (iii) in each group, fitness parameters (fecundity of G_1_ snails and survival of G_2_ offspring) were monitored during one month.

**Results:**

The sexual maturity (age at first egg-laying) was reached, on average, at 63.9 (sd: 3.0) and 103.7 (sd: 36.6) days for *B. pfeifferi* and *B. camerunensis*, respectively. Copulation was observed in all paired individuals in both species. In *B. pfeifferi*, the fecundity (number of egg capsules and eggs) of young G_1_ individuals and survival of G_2_ offspring on D_0_ and D_8_ were similar between selfing and outcrossing individuals, and a very low inbreeding depression (0.063) was observed. In *B. camerunensis*, the fecundity of outcrossed individuals was significantly higher than that of selfed individuals. The hatching rate was significantly higher and the incubation time significantly shorter for cross-fertilized eggs as compared with self-fertilized eggs, and a high inbreeding depression (0.71) was observed.

**Conclusion:**

These findings may explain the high adaptability to more diverse and inconstant habitats, as well as the better compatibility of *B. pfeifferi* to *S. mansoni* compared with *B. camerunensis*, and may support the sustainability of *S. mansoni* life cycle where this intermediate host prevails.

**Electronic supplementary material:**

The online version of this article (doi:10.1186/s13071-015-1285-4) contains supplementary material, which is available to authorized users.

## Background

Hermaphrodism has been reported in 21 phyla of the Animal Kingdom, and self-fertilization is known in ascidians, cestodes, cnidarians, nematodes, trematodes, mollusks and vertebrates [1]. Fresh water pulmonate gastropods, especially basommatophorans, are the best studied group of mollusks [2–5]. Indeed, these snails represent an excellent model for mating system studies since (i) they are somehow easy to breed in the laboratory owing to their short life cycles (about 2–10 generations can be produced each year), and (ii) they are simultaneous hermaphrodites, thus able to reproduce through either self- or cross-fertilization [3, 6]. Cross-fertilization requires copulation during which one individual acts as a male while the other acts as a female. Female-acting fertilized individuals may store and use foreign sperm for several weeks after copulation without additional pairing [7], as has been previously reported in preferential outbred species such as *Biomphalaria glabrata* [8] or *Bulinus globosus* [9]. In the absence of mates, mature snails can either wait for the arrival of a hypothetical partner and pay for the cost of this delay (the increased risk of dying without reproducing), or self-fertilize their eggs and experience inbreeding depression [10, 11]. Inbreeding or self-fertilization depression refers to the relative reduction in fitness of inbred offspring as compared to outbred offspring, and may be used to study the mating system evolution among snail species [9, 11–13]. It was shown that preferential outcrossing species generally exhibit a high inbreeding depression [11] as has been observed in *Lymnaea peregra* [12], *Physa acuta*, *Bulinus globosus*, *Biomphalaria glabrata* [11] and *Drepanotrema deprissimum* [14]. However, self-crossing species such as *Bulinus truncatus*, *Bulinus forskalii*, and *B. pfeifferi* generally exhibit a low inbreeding depression [11].

The present study focuses on two gastropod species, *B. pfeifferi* and *B. camerunensis* that act as intermediate hosts of the trematode *Schistosoma mansoni*, and for which very few data regarding their life history traits are available. Both species are hermaphroditic fresh water snails, belonging to the Planorbidae family. *B. pfeifferi* dwells in a variety of more or less permanent water bodies including streams, irrigation channels and dam lakes [15–18]. Flooding and drought are responsible for the important fluctuations in population densities of this snail species, thus leading to bottlenecks and recolonization events [12, 18]. As a consequence, limited neutral variability within populations and fairly large genetic differentiation among populations can be observed [17–19]. *B. pfeifferi* was described as a prior selfing species (selfing initiated prior to any outcrossing), very few copulations being observed in paired snails [20]. Genetic analyses at both family and population levels have indicated high selfing rates in *B. pfeifferi* [18, 21, 22]. *Biomphalaria camerunensis species* is distributed from Ghana eastwards to Central African Republic, and southwards to lower Democratic Republic of Congo (former Zaire) [16]. In Cameroon, *B. camerunensis* is confined to the southern equatorial climatic zone [23], and was never found in the same water collection with *B. pfeifferi*. It dwells in a variety of permanent waters including rivers, streams, lakes, swamps, and sometimes in temporary pools. *B. camerunensis* was considered as a preferential outbred species since population genetic studies have revealed heterozygote deficiency [24].

Despite these previous studies, an extensive knowledge of the mating systems of these two snail species may be helpful to master the transmission heterogeneity observed in intestinal schistosomiasis in Cameroon. In fact, *B. pfeifferi* is the main intermediate host of this trematode whereas *B. camerunensis* only plays a minor role in the transmission [16]. This knowledge can furthermore contribute to the development of strategies to control *Schistosoma mansoni*. Indeed, it was demonstrated that *Biomphalaria tenagophila* Taim lineage is highly resistant to *S. mansoni*, this innate defense system being a dominant character in cross-breedings with susceptible strains [25]. Thus, mastering the mating system of these intermediate hosts appears to be of prime importance in generating offspring resistant to the parasites after introduction of snail competitors [26]. To this end, a comprehensive study of the mating system of both *Biomphalaria* species was performed by monitoring the life history traits and evaluating the fitness parameters of isolated and paired snails under laboratory conditions.

## Methods

### Snail collection and rearing conditions

The two snail species included in this study were collected in two sites, both located in the Centre Region of Cameroon. A total of 28 wild *B. camerunensis* individuals were collected, in November 2012, in the Mounassi pond (4°12’14.1” N and 11°35’00.2” E) in the Minkama III village (near Obala city). The water conductivity of the pond was 230 Siemens and its pH equal to 7.30. Also, 54 wild *B. pfeifferi* individuals were sampled, in March 2013, in a piscicultural lake in the Etokos quarter (3°48’33.3” N and 11°23’06.0” E) located in the Yaoundé city. The conductivity and pH of the water of the lake were 162 Siemens and 7.12, respectively. All the snails collected were brought alive to the snail rearing room at the Faculty of Science of the University of Yaoundé 1, and maintained at 26 ± 1 °C throughout the experiment, under a 12 L/12D photoperiod. The conductivity of rearing water was 196 Siemens, and its pH equal to 6.96. During the rearing, snails were fed *ad libitum* with dried lettuce. Water and lettuce were changed every day in rearing boxes containing very young snails, or every two days in those with juveniles and adults. Small floating pieces of polystyrene were introduced in the rearing boxes for egg capsules’ laying.

### Experimental plan

The experimental design of the present study is given as supplementary material [Additional file 1: Figure S1]. In the laboratory, wild snails (G_0_) from each species were kept together in a 1.5 L plastic box for acclimatization. Two days later, 15 mature individuals from each species were randomly chosen and isolated in 100 mL boxes. Two egg capsules were collected for each individual and incubated separately in 100 mL boxes. After hatching, juveniles of the first generation (G_1_) were maintained in rearing boxes for approximately three weeks. Three to four G_1_ juveniles, aged 26 to 30 days (i.e. before sexual maturity) were chosen at random from each box, and a sample of 120 G_1_ snails constituted for each species. These G_1_ individuals were isolated and reared until sexual maturity (first-egg-laying) was reached. The first individuals reaching sexual maturity were assigned to one of the two treatments, selfing (T_1_) and outcrossing (T_2_). Indeed, 40 *B. pfeifferi* and 33 *B. camerunensis* individuals were kept isolated throughout the experiment, and therefore self-fertilized (T_1_); 40 *B. pfeifferi* were paired together (20 couples) and 36 *B. camerunensis* were paired together (18 couples) during three successive days (see more details below) after which they were isolated again (T_2_). After the three days of copulation, two egg capsules were collected for each snail from each treatment for the estimation of hatching and survival (over a month) rates for individuals of the second generation (G_2_).

### Assessment of life-history traits

For each individual snail, the age at sexual maturity (age at first egg-laying) was recorded, and the numbers of egg capsules as well as eggs laid over 30 days, according to the type of treatment, were counted using a binocular magnifying glass. After the three days of pairing, one capsule was collected per snail to estimate the juvenile survival rate. To do this, the number of living embryos, the incubation time (estimated in days), the number of hatching individuals (from the first day of survival, that is on D_0_), as well as the number of juveniles surviving at 8 (D_8_), 16 (D_16_) and 30 (D_30_) days after hatching were recorded. Inbreeding depression was calculated in each group using the following formula:

$$ \mathrm{d}=1-\left(\mathrm{W}\mathrm{s}/\mathrm{W}\mathrm{c}\right),\mathrm{where}\kern0.5em \mathrm{W}=\kern0.5em {\mathrm{v}}^{*}\mathrm{f} $$

In this formula, W_s_ and W_c_ stand for the selective value in self-fertilization and cross-fertilization, respectively; v is the hatching rate or the viability of G_2_ snails over 30 days, and f the mean number of eggs laid by G_1_ snails over 30 days. The inbreeding depression was assessed using an easy-to-use indirect method as previously demonstrated in other Basommathophorans [11] and in plants [27]. Its estimation was possible since cross-fertilization was demonstrated in paired *B. pfeifferi* individuals [28]. Since inbreeding depression is usually larger at the earlier stage of the life cycle, apparent inbreeding depression, taking into account only hatching and early survival of juveniles, was computed [29].

### Statistical analyses

Statistical analyses were performed using the software *PASW Statistics* 18 (SPSS Inc., Chicago, IL, USA). A normality test was first performed to assess the distribution of each quantitative parameter. Then, the mean number of egg capsules, eggs per capsule laid, copulation and average copulation duration were compared between isolated and paired individuals using the parametric Student t-test, and the number of hatched eggs as well as the incubation time between the two groups were compared using the non-parametric Man Whitney test. The hatchability (evaluated on Day 0) and survival of the offspring (assessed on D_8_, D_16_ and D_30_) were compared using the Chi-square test.

## Results

### Age at sexual maturity

Sexual maturity was reached between 59 and 71 days (mean: 63.9; standard deviation (sd): 3.0), and between 60 and 175 days (mean: 103.7; sd: 36.6) for *B. pfeifferi* and *B. camerunensis*, respectively. The age at sexual maturity was reached earlier for *B. pfeifferi* individuals as compared to *B. camerunensis* individuals (*p* < 0.005).

### Selfing estimates

All *B. pfeifferi* isolated individuals survived and laid egg capsules during the 32 weeks of the performance of the experiment. However, among the 119 *B. camerunensis* isolated individuals, 17 (14.3 %) died within the 32 weeks of the performance of the experiment, 69 (58.0 %) laid at least one egg capsule during this period, whereas 33 (27.7 %) never laid any egg capsule. The number of egg capsules laid was similar between the two snail species (*p* = 0.95), whereas the mean number of eggs was higher in *B. pfeifferi* than in *B. camerunensis* individuals (*p* < 0.0001) (Table 1). Although the incubation time was similar between the two species, the hatching rate was significantly higher in *B. pfeifferi*, and survival of G_2_ individuals was higher in *B. camerunensis* (Table 1).

**Table 1:** Fecundity and survival estimates for isolated (T1) and paired (T2) individuals from *Biomphalaria pfeifferi* and *Biomphalaria camerunensis* populations

Parameters	Snail species	T_1_	T_2_	*p*-value
Number of G_1_ individuals	*B. pfeifferi*	35	34	
	*B. camerunensis*	30	30	
Mean number of egg capsules (sd)	*B. pfeifferi*	19.0 (4.1)	19.3 (3.7)	0.732
	*B. camerunensis*	18.9 (12.6)	27.9 (11.9)	0.010^*^
Mean number of eggs (sd)	*B. pfeifferi*	363.2 (66.0)	366.6 (72.9)	0.840
	*B. camerunensis*	121.2 (95.8)	315.2 (183.8)	<0.0001^*^
Number of incubated capsules	*B. pfeifferi*	32	30	
	*B. camerunensis*	36	42	
Average incubation time (sd)	*B. pfeifferi*	9.6 (0.5)	9.4 (0.8)	0.202
	*B. camerunensis*	10.0 (1.7)	8.2 (0.9)	<0.0001^*^
Hatching rate (95 % CI)	*B. pfeifferi*	87.2 (84.2–89.7)	92.3 (89.5–94.4)	0.5323
	*B. camerunensis*	68.8 (63.6–73.5)	92.3 (89.9–94.3)	<0.0049^*^
Number of G_2_ offspring	*B. pfeifferi*	30	30	
	*B. camerunensis*	36	36	
G_2_ survival rate at D_8_ (95 % CI)	*B. pfeifferi*	72.6 (68.4–76.5)	67.4 (62.8–71.6)	0.0848
	*B. camerunensis*	94.3 (90.5–96.6)	82.5 (78.8–85.7)	0.0001^*^
G_2_ survival rate at D_16_ (95 % CI)	*B. pfeifferi*	64.2 (59.7–68.5)	50.1 (45.5–54.8)	0.0001^*^
	*B. camerunensis*	85.5 (80.4–89.5)	79.7 (75.9–83.2)	0.648
G_2_ survival rate at D_30_ (95 % CI)	*B. pfeifferi*	54.1 (49.5–58.6)	39.2 (34.8–43.9)	0.0001^*^
	*B. camerunensis*	82.5 (77.0–86.9)	76.7 (72.7–80.3)	0.0838

*sd: standard deviation;* 95 % *CI* 95 % confidence interval; ^*^ *the difference is significant*

### Copulation metrics

Copulations were observed in all 17 pairs of virgin *B. pfeifferi* individuals and in 15 pairs of virgin *B. camerunensis* individuals (Table 2)*.* For *B. pfeifferi*, the mean number of copulations was similar between the first and the second day of pairing (*p =* 0.064), but was significantly lower on the third day compared with the first two days of pairing (*p* < 0.0001). As for *B. camerunensis*, the mean number of copulations over the three days of pairing was similar between the first and the second day (*p* = 0.634), but significantly lower on the third day compared with the first two days of pairing (*p* < 0.006) (Table 2). Regarding the duration of copulations, a significant decreasing trend was observed between the first and the third day of pairing for *B. pfeifferi* (*p* < 0.0001) (Table 2). For *B. camerunensis*, the duration of copulations also gradually decreased during the three days of pairing, lasting about five hours the first day, and only fifteen minutes afterwards for most of the copulations. Although the mean number of copulations per individual and per day of pairing was significantly higher in *B. pfeifferi* compared with *B. camerunensis* (Table 2), the total duration of copulations over the three days of pairing was higher in *B. camerunensis* than in *B. pfeifferi.*

**Table 2:** Frequency and duration of copulations in *Biomphalaria pfeifferi* and *Biomphalaria camerunensis* populations

Parameters	Snail species	No couples	Mean	sd of mean
Copulation frequency at first day of pairing	*B. pfeifferi*	15	1.9	0.7
	*B. camerunensis*	15	1.9	0.7
Copulation frequency at second day of pairing	*B. pfeifferi*	17	2.5	0.5
	*B. camerunensis*	15	1.7	0.7
Copulation frequency at third day of pairing	*B. pfeifferi*	17	1.2	0.7
	*B. camerunensis*	15	1.0	0.7
Copulation frequency over three days of pairing	*B. pfeifferi*	17	6.6	1.2
	*B. camerunensis*	15	4.6	1.3
Copulation duration at first day of pairing	*B. pfeifferi*	17	5.6	0.7
	*B. camerunensis*	15	5.3	2.0
Copulation duration at second day of pairing	*B. pfeifferi*	17	3.8	0.9
	*B. camerunensis*	15	4.1	1.6
Copulation duration at third day of pairing	*B. pfeifferi*	17	1.7	1.1
	*B. camerunensis*	15	2.0	1.3
Copulation duration over three days of pairing	*B. pfeifferi*	17	11.1	1.9
	*B. camerunensis*	15	11.4	3.5

*No: number of; sd: standard deviation*

### Outcrossing estimates

**Fig. 1:**
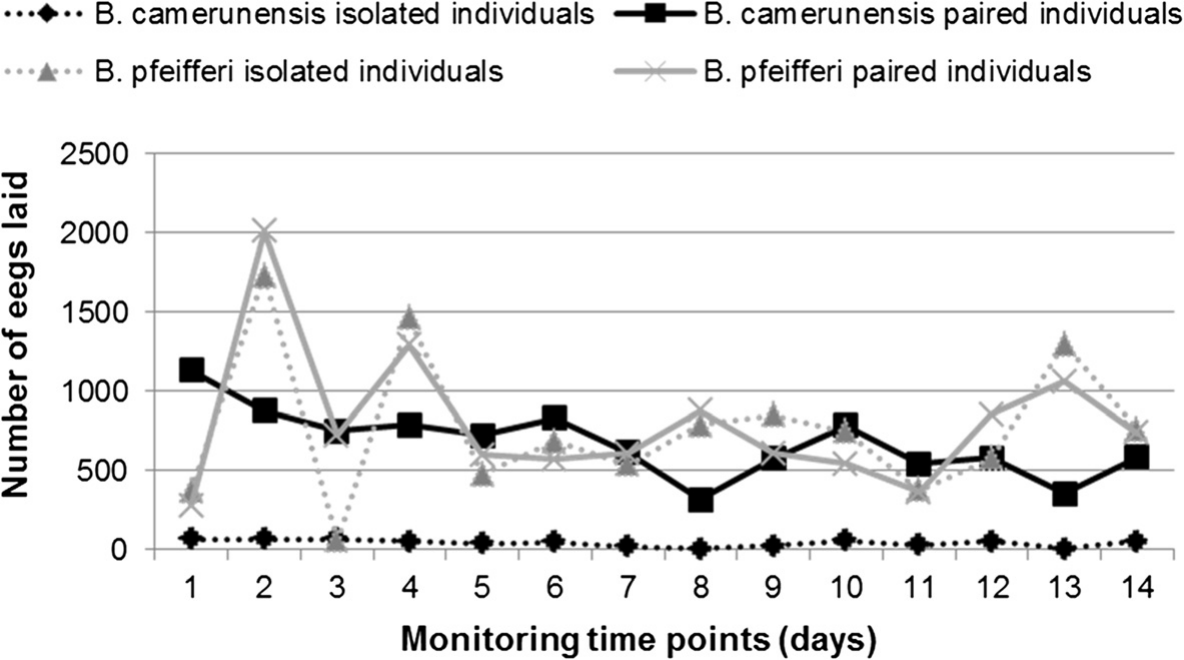
Egg laying dynamics for isolated and paired individuals from *Biomphalaria pfeifferi* (grey lines) *and Biomphalaria camerunensis* (dark lines) populations. Dotted lines represent isolated individuals, and solid lines represent paired individuals

**Fig. 2:**
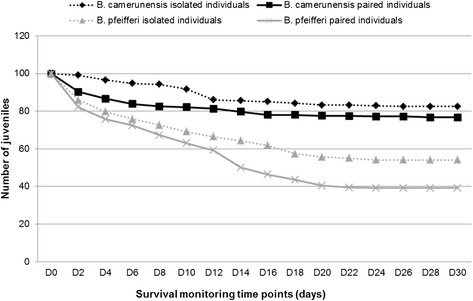
Thirty-day survival trends in isolated and paired individuals from *Biomphalaria pfeifferi* (grey lines) and *Biomphalaria camerunensis* (dark lines) populations. Dotted lines represent isolated individuals, and solid lines represent paired individuals

*B. camerunensis* egg production greatly increased within 6 days just after copulation before gradually decreasing, a maximum of 1,131 eggs being produced by paired individuals two days after copulation (Fig. 1; Table 1). No significant association was found between the number of copulations of paired *B. camerunensis* and both the number of egg capsules (r = 0.225; *p* = 0.233) and eggs (r = 0.221; *p* = 0.241). The mean number of egg capsules was significantly higher in *B. camerunensis* compared with *B. pfeifferi* (*p* = 0.001), whereas the mean number of eggs was similar between both species (*p* = 0.160). The offspring survival rate was, in general, higher in *B. camerunensis* than in *B. pfeifferi*, the survival slope revealing higher mortality on D_2_, with subsequent decrease until D_12_ before stabilizing on D_16_ (Fig. 2). A positive correlation was found between mortality and density of snails in the rearing boxes on D8 (r = 0.625; *p* < 0.0001), D14 (r = 0.620; *p* < 0.0001), and D30 (r = 0.719; *p* < 0.0001).

### Comparison between the two treatments and inbreeding depression

For *B. pfeifferi*, the number of egg capsules was not significantly different between inbred (mean: 19.0; sd: 4.1) and outbred (mean: 15.3; sd: 3.7) (*p* = 0.732) individuals. A similar trend was observed for the mean number of eggs, with no significant difference between inbred (mean: 363.2; sd: 66.0) and outbred individuals (mean: 366.6; sd: 72.9) (Fig. 1). Incubation time and hatchability were similar between the two treatments (Table 1). Although survival rate was similar between selfed and outcross offspring on D_8_ (Chi-square: 3.0; *p* = 0.08), significant differences were found on D_16_ (Chi-square: 18.2; *p* < 0.0001) and D_30_ (Chi-square: 19.81; *p* < 0.0001), inbred offspring having a better survival rate than their outbred counterparts.

For *B. camerunensis*, the number of egg capsules as well as the number of eggs laid was significantly lower in inbred than in outbred individuals (*p* < 0.01) (Table 1). The hatching rate of G_2_ offspring was significantly higher in outbred eggs (92.3 %) compared with inbred eggs (68.8 %) (Chi-square: 7.9; *p* = 0.005). Also, these outbred eggs hatched faster than the inbred ones (Table 1). The survival rate of G_2_ offspring from inbred G_1_ individuals, evaluated on D_8_, was significantly higher than that of G_2_ offspring from outbred G_1_ (Chi-square: 18.0; *p* = 0.0001).

The self-fertilization (inbreeding) depression using the survival on D_0_ (in such a way as to minimize the effect of density) and the survival on D_30_ was much higher in *B. camerunensis* (d = 0.71 and d = 0.53, respectively) than in *B. pfeifferi* (d = 0.06 and d = - 0.26, respectively).

## Discussion

### Rearing and natural living conditions of snails

The rearing conditions in the laboratory were slightly different from the natural living conditions of snails. Furthermore, these conditions, especially the pH, seemed to be more favourable to *B. pfeifferi* than to *B. camerunensis* culture. However, numerous previous studies have shown that gastropod snails are tolerant to most water physicochemical parameters [30]; it was particularly demonstrated that the pH of water has little effect on mollusk populations, gastropods snails being able to tolerate all pH values, with a preference to standard values (between 6 and 9) [31].

### A plastic life-history trait: the age at sexual maturity

Reproduction of inbred individuals in *B. pfeifferi* population was initiated at about 63 days, later than previously reported in another Cameroonian population [28] but earlier than previously reported in Ivory Coast [20]. These differences in the age at sexual maturity can be explained by the large genetic differentiation among *B. pfeifferi* populations [17–19]. This finding is supported by the habitat openness (that reflects environmental stochasticity), the prevalence of the parasitic trematode *S. mansoni* and historical demography (colonization and subsequent bottlenecks). *B. camerunensis* individuals began laying eggs later than *B. pfeifferi* individuals, probably due to a delay resulting from a waiting time exhibited by isolated *B. camerunensis* individuals after reaching sexual maturity. Indeed, in preferential outcrossing species such as *B. camerunensis*, instead of laying bad eggs, isolated individuals tend to delay their reproduction to wait for the arrival of a mate [10]. This late egg-laying might lead to the overestimation of the age at sexual maturity for preferential outcrossing species. Also, the late age at first egg-laying in *B. camerunensis* allows the development of only 2 to 3 generations each year and may explain why the biology of this snail species is still less studied. On the contrary, the early age at first egg-laying exhibited by *B. pfeifferi* population might be considered as an advantageous feature for a fresh water snail population living in transient habitats.

### Copulatory activity

Contrary to previous reports in high selfing populations like *B. pfeifferi* [20, 32], an important number of copulations was observed in our study. This could be explained either by differences in the experimental designs or by the high diversity of life history traits exhibited by *B. pfeifferi* populations [17–19]. In our study, individual snails were kept isolated during six weeks and paired after reaching sexual maturity, whereas in other experimental designs, individuals were isolated only two weeks prior to pairing, the latter starting before sexual maturity [20]. Virgin individuals, isolated since they hatched, might be eager to copulate as soon as they are paired as previously demonstrated either in selfing species like *B. truncatus* [33] or in outcrossing species like *B. globosus* [34, 35] and *B. glabrata* [36]. Despite a very low value of inbreeding depression in *B. pfeifferi* populations, copulations were observed in all pairs, indicating that copulation has no substantial influence on fecundity as has been previously shown in selfing species. Indeed, even when partners are available and copulation occurs, a large fraction of eggs from preferential selfing species self-fertilized [11] and only a small fraction of offspring then derive from outcrossing. Since previous studies using genetic markers had already demonstrated successful sperm transfer in *Bulinus truncatus* [33] and in *Biomphalaria pfeifferi* [22, 28], no genetic analysis was performed in the framework of our study. More investigations using molecular markers such as microsatellites would have been useful to assess the real fraction of individuals of this species that used foreign sperm. Also, the time and the number of copulations per day decreased over time as previously reported in *B. forskalii*, another preferential selfing species [13]. Once again, the experimental conditions can explain this observation.

Copulations were reported in all *B. camerunensis* pairs and the courtship behavior began within 30 min after pairing virgin individuals. These observations suggest that these individuals have a high propensity to copulate, a dominant mating system feature in outcrossing species [11]. In our study, the number of copulations was low than in *B. glabrata* [32] and in *P. acuta* [37], and might be explained by the design of our study. Also, no correlation was observed between the number of copulations and the fecundity, meaning that a single copulation may be enough to boost fecundity.

### Mating system parameters

All isolated and virgin *B. pfeifferi* individuals started to lay eggs as soon as they reached sexual maturity. They also regularly produced egg capsules with fertile eggs, with a high hatching rate and subsequent survival. These observations demonstrate that *B. pfeifferi* individuals did not suffer from strong inbreeding, suggesting that selfing is the preferential mating system for this population. This was not surprising and confirms previous findings reporting the characterization of prior selfing (selfing initiated prior to any outcrossing) in *B. pfeifferi* [20]. In addition, no difference in fecundity (number of egg capsules and the number of eggs) was found between isolated and paired individuals, suggesting that pairing did not significantly affect the selfed mating system previously adopted by isolated individuals. Similar results have been observed in *B. globosus* from Ivory Coast, and *B. forskalii* from Cameroon [9, 13]. It also appears that the presence of a mate partner does not significantly affect the development (incubation time) and the hatchability of eggs since both parameters were similar between the two treatments. However, the survival rate in G_2_ offspring from selfing G_1_ was significantly higher than in G_2_ from outcrossed G_1_. This result differed from that obtained in other *B. pfeifferi* populations where the survival rate of the selfing and the outcrossing groups were similar [20]. This might be explained by the higher hatching rate of G_1_ egg capsules in outbred (92.3 %) than in inbred (87.2 %). As a consequence, a high offspring density was observed in boxes dedicated to outcrossing, and might explain the higher mortality rate observed. As expected, a limited inbreeding depression was observed in this study, indicating that selfing might be the preferential mating system in *B. pfeifferi* as previously suggested using isoenzymes [21]. In pulmonate snails, reproduction by selfing seems easy from a functional point of view. Both sperm and ovules are produced in a single reproductive organ, the ovotestis, and self-fertilization occurs within the hermaphroditic part of the reproductive tracts, while cross-fertilization requires copulation. Beyond this functional reason, the important reduction of the population size during some seasons can explain the selection of selfing as the preferential mating system.

Contrary to *B. pfeifferi* population, only 57.1 % of *B. camerunensis* isolated individuals laid eggs before dying. Also, isolated individuals produced irregularly-shaped egg capsules containing few (or no) eggs. These egg capsules were smaller than those produced by the same snail after copulation. These observations suggest that these individuals markedly suffered from strong inbreeding, and indicate that this species prefers a biparental reproduction as was previously demonstrated in another obligate outcrosser *Drepanotrema depressissimum* [14]. Regarding the fecundity of these individuals, it appears that the mean numbers of egg capsules and eggs per snail were significantly higher in outbred than in inbred individuals. In cross fertile species *L. peregra* and *Bulinus cernicus,* no difference in egg capsules was found between the two treatments, but the mean number of egg (either per snail or per day) significantly increased after copulation [12, 38]. A positive correlation (r = 0.830; *p* < 0.0001) was found between the number of egg capsules and the number of eggs, meaning that a snail producing more egg capsules also lays more eggs after copulation. This high fecundity can be explained by the transfer of sperm as it was demonstrated in *L. stagnalis* [34]. In the course of our study, egg production greatly increased within a 6-day period just after copulation, and subsequently decreased. Similar trends were reported in *B. globosus* where isolated individuals switched to cross-fertilization less than 6 days after copulation, and were able to conserve the foreign semen for up to 11 days [9]. Since the foreign semen gradually decreased during three to five weeks after copulation, one can hypothesize that it was used for reproduction [36, 39, 40]. In spite of a higher hatchability in outcrossed egg capsules as compared to selfed egg capsules, no significant difference was found in the survival of G_2_ juveniles. It was previously observed in other snail species (*L. peregra* and *D. depressissimum*) that survival rate in cross-fertilized juveniles was significantly higher than in the selfed ones. The high mortality rate in G_2_ offspring deriving from outcrossing G_1_ individuals might be explained by the high offspring densities in the rearing boxes. Indeed, an average of 13 individuals were observed in each box, whereas only six individuals were observed, on average, in each box containing G_2_ offspring derived from selfing G_1_ individuals. Another explanation might be the very short development of eggs in outcrossed egg capsules as a consequence of a short incubation time. As expected, our results indicated stronger differences between the selective values in self-fertilization than in cross-fertilization, with an inbreeding depression value lower than that obtained in *L. peregra* during the same time period (one month) [12]. On the contrary, this value was higher than the average apparent inbreeding depression displayed by *B. glabrata*, another preferential outcrosser species [11, 41]. It was hypothesized that the inbreeding depression in outcrossing populations is higher than one half [2, 42, 43]. Our results fit with this general prediction, and confirm that *B. camerunensis* is a preferential outcrosser species.

## Conclusions

Although genetic markers were not used to ascertain that the eggs produced after pairings were the result of outcrossing and not a continuation of selfing, the present study tends to indicate, from phenotypical evidence, that the preferential mating system of *B. pfeifferi* is selfing. This result might explain why this species colonizes more diverse and inconstant habitats than *B. camerunensis* whose preferential mating system is outcrossing. In addition to that invasive ability, *B. pfeifferi* is suggested to be more compatible to *S. mansoni* than *B. camerunensis*, and then this mating system could favor the sustainability of *S. mansoni* life cycle where this intermediate host is present.

## Additional file

Additional file 1:
**Study design.** (TIF 120 kb)

## Abbreviations

G_0_: wild individuals
G_1_: first generation
G_2_: second generation
sd: standard deviation
95 % CI: 95 % confidence interval
