# Supplementary figures and images for: Analysis of mating system, fecundity, hatching and survival rates in two Schistosoma mansoni intermediate hosts (Biomphalaria pfeifferi and Biomphalaria camerunensis) in Cameroon
Source: Parasit Vectors. 2016 Jan 6;9:10. doi: 10.1186/s13071-015-1285-4 (PMC4702333; doi:10.1186/s13071-015-1285-4)

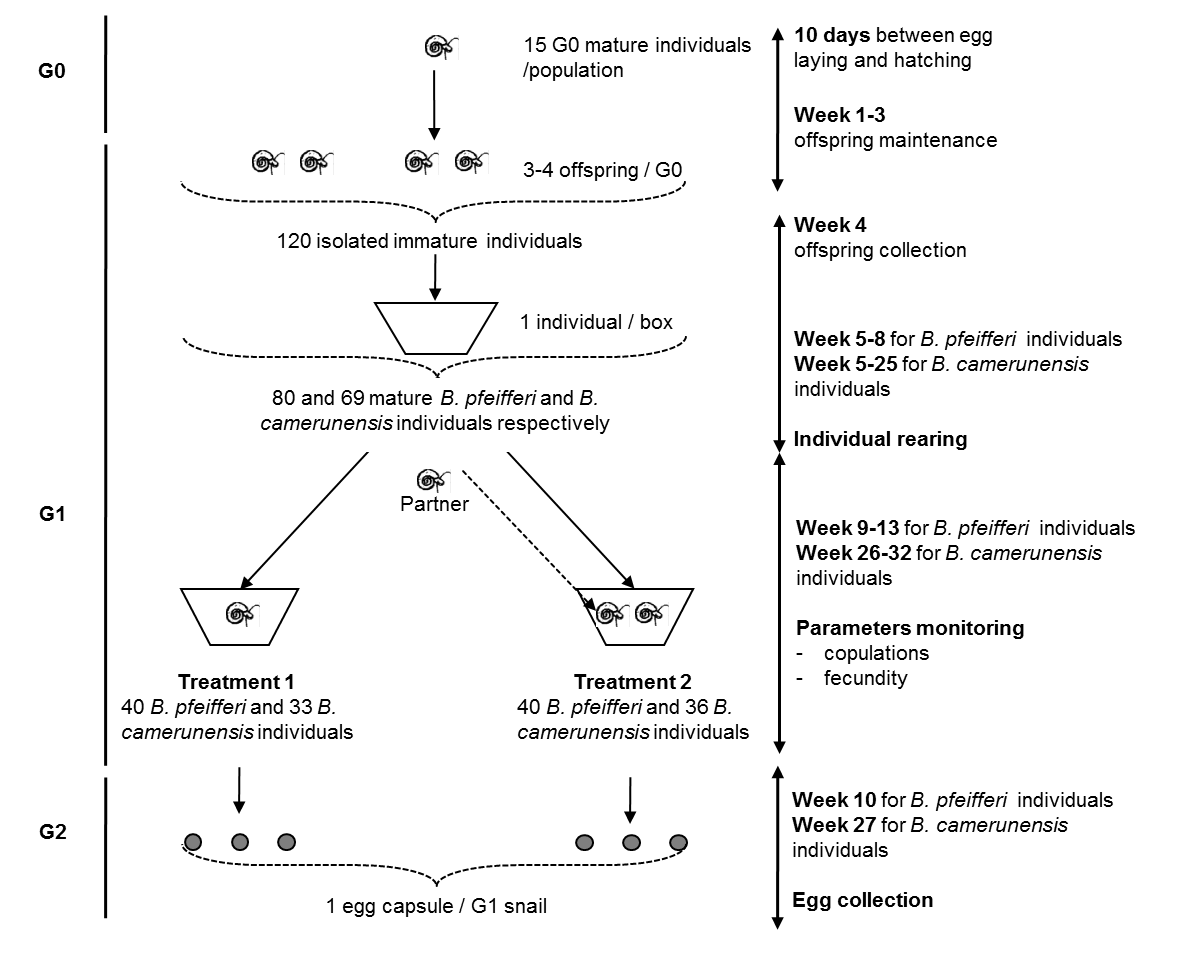

Supplement: Additional file 1: — Study design. (TIF 120 kb) [file 13071_2015_1285_MOESM1_ESM.tif]
